# Epithelial–Mesenchymal Transition-Mediated Tumor Therapeutic Resistance

**DOI:** 10.3390/molecules27154750

**Published:** 2022-07-25

**Authors:** Zhimin Xu, Yingxin Zhang, Huanyan Dai, Bing Han

**Affiliations:** 1 Department of Oral and Maxillofacial Surgery, School and Hospital of Stomatology, Jilin University, Changchun 130021, China; xuzhimin@jlu.edu.cn (Z.X.); daihy20@mails.jlu.edu.cn (H.D.); 2 Department of Oral Emergency, Hospital of Stomatology, Jilin University, Changchun 130021, China; tongtong31029@jlu.edu.cn

**Keywords:** EMT, chemoresistance, radioresistance, targeted therapy, immunity therapy

## Abstract

Cancer is one of the world’s most burdensome diseases, with increasing prevalence and a high mortality rate threat. Tumor recurrence and metastasis due to treatment resistance are two of the primary reasons that cancers have been so difficult to treat. The epithelial–mesenchymal transition (EMT) is essential for tumor drug resistance. EMT causes tumor cells to produce mesenchymal stem cells and quickly adapt to various injuries, showing a treatment-resistant phenotype. In addition, multiple signaling pathways and regulatory mechanisms are involved in the EMT, resulting in resistance to treatment and hard eradication of the tumors. The purpose of this study is to review the link between EMT, therapeutic resistance, and the molecular process, and to offer a theoretical framework for EMT-based tumor-sensitization therapy.

## 1. Introduction

The epithelial–mesenchymal transition (EMT) is a cellular process that often occurs during normal or pathological processes. It is essential in embryonic development and wound healing and is crucial for tumor growth [1]. Many factors trigger EMT in tumor cells, such as tumor microenvironment [2,3] and inflammatory cytokines [4]. Indeed, EMT is associated with increased cancer stem cells, inadequate anti-tumor immunity, and resistance to oncology therapies. Consequently, EMT is an essential component of the invasion-metastasis cascade. Despite the diversity of cancer types, the EMT process in patients with low-grade gliomas might have significant prognostic consequences [5].

When tumor cells undergo EMT, they lose their epithelioid characteristics and exhibit poor intercellular adhesion and polarity, resulting in enhanced tumor cell motility and invasion [6]. EMT is also associated with abnormal molecular expression patterns, including decreased epithelioid marker expression, increased mesenchymal marker expression, and EMT-related transcription factors. Given that epigenetic processes such as DNA methylation and chromatin changes may impact EMT, a complex but reversible process, they are required to maintain its regulation. Loss of the cell cycle regulator p21 has been associated with increased EMT characteristics and a rise in ZEB1, the master EMT transcription factor [7,8,9].

Tumor cells adapt to cytotoxicity and develop therapeutic resistance due to EMT-induced anti-apoptotic abilities, improved DNA damage repair, and a modified drug metabolism pathway [10]. At present, distinct EMT processes have been seen in tumor tissues and cells that are resistant to chemotherapy or radiation, suggesting that EMT is intrinsically linked to tumor treatment resistance [11,12,13]. In this review, we addressed the characteristics of EMT; further discussed tumor therapeutic resistance, discussed EMT-mediated therapeutic resistance and its molecular mechanism, and appraised the level of research on EMT and tumor therapeutic resistance.

## 2. Overview

Epithelial cells undergo a shift to mesenchymal cells during EMT. Their gel-cultured lens epithelial cells lose their polarity, extend their pseudopodia, and acquire mesenchymal features, in a process known as the epithelial–mesenchymal transition. EMT often develops due to physiological or pathological processes occurring inside the human body. According to the biological setting in which EMT occurs, it may be categorized as follows:

Type 1 EMT is a term that refers to an EMT that occurs during embryogenesis and organ development [14].

Type 2 EMT is defined as an EMT that responds to wound healing, tissue regeneration, or organ fibrosis [15,16].

Type 3 EMT refers to the conversion of epithelioid cells to mesenchymal cells that occurs during the development of malignant tumors and is associated with treatment resistance and distant metastasis [17,18]. This review discusses the phenotypic and molecular processes underlying the tumor treatment resistance generated by type 3 EMT.

## 3. Therapeutic Resistance of Tumors

Surgery, chemotherapy, radiation, and targeted molecular therapy are the principal therapies for solid tumors. These strategies have been shown to successfully remove, damage, and induce cell death, hence resulting in disease remission. A combination of one or more treatments may be used depending on the type of tumor and tumor stage. Patients with advanced cancers typically miss out on major surgery due to the extra chemotherapy, radiation therapy, and targeted therapy treatments required. While some degree of control is achievable, cancer chemotherapeutic drug resistance, radiation resistance, and targeted treatment resistance contribute to the failure to produce a radical curative effect, posing a substantial barrier to cancer eradication. Tumor treatment resistance is a complex process that is impacted by various factors, including the tumor microenvironment, non-coding RNA, and EMT [19,20,21,22,23].

Due to the heterogeneity of tumors, treatment-resistant mutations are filtered out throughout therapy, or latent, initially drug-resistant tumor stem cell subsets are awakened, resulting in treatment failure and tumor recurrence or metastasis. Additionally, tumors in the same area are varied in terms of cell type, clinical stage, and degree of differentiation. Furthermore, homogeneous tumors demonstrate significant molecular variety, including differences in gene expression patterns, network regulation, and mutation profiles [24,25]. The heterogeneity of patients with the same kind of tumor is reflected in the inconsistency of the changed gene spectrum and biological features across cells, reflecting malignant tumors’ enormous complexity and diversity during their lifetime. More importantly, tumor heterogeneity may contribute to tumor cell resistance to treatment by modifying epigenetic factors such as messenger RNA, the transcriptome, and the proteome. Simultaneously, tumor stem cells are related to resistance to cancer treatment [26,27,28]. Most cancer treatments, including anticancer drugs, are modulated by innate processes that function as a defense against environmental toxins. Defense mechanisms include drug efflux mediated by ATP binding box (ABC) transporters. The ABC transporters is the most common transmembrane transporter for drug efflux. The human genome consists of seven subfamilies of ABC genes (ABCA-ABCG). ABCB1, ABCC1, and ABCG2 are essential for developing cancer chemotherapy resistance (MDR) [29,30]. ABCB 1 was highly expressed on the surface of some tumor cells. In this case, the cytoplasm is able to pump the chemotherapeutic substance of the intracellular ABCB 1 substrate out of the cytoplasm. Overexpression of ABCB 1 on the cytoplasm surface further amplifies this response. This is one of the main mechanisms of reduced intracellular drug accumulation and the generation of multidrug resistance in tumors [31]. ABCG2, named Breast cancer resistance protein, is the main drug efflux transporter that leads to resistance in breast cancer. ABCG2 is thought to be a marker of cancer stem cells (CSCs) in some cancers and is to blame for the side-population effect. It can move medicines with either a positive or a negative electrical charge, starting with chemotherapeutic agents. ABCG2 substrate chemotherapeutic substances in tumor cells can be transferred to the outside of tumor cells, which reduces the sensitivity of tumor cells to chemotherapy drugs. This leads to the emergence of multidrug resistance [32,33]. For instance, the expression of the ABC transporters was similarly increased after TGF-β or Twist in breast cancer cells, and zeb1 exhibited the reverse-transition EMT and its resistance to the virobistar [34,35]. Similarly, it was reported that the transcription factors that govern EMT might be utilized to regulate the high expression of the ABC transporter protein by regulating the transmission-drug pump gene, therefore decreasing the drug concentration in the cell and making the cells susceptible to drug treatment. The EMT transcription factor may also be modulated by interfering with the apoptotic pathway to render tumor cells drug-resistant [36,37].

Recent advances in the tumor microenvironment study have shown that the tumor microenvironment is intricately linked to tumor treatment resistance. Indeed, the tumor microenvironment is composed of cellular and noncellular elements, including tumor cells, fibroblasts, endothelial cells, and immune cells [38,39,40]. The change from fibroblasts into tumor-associated fibroblasts in the tumor microenvironment may result in tumor resistance to chemotherapy. The infiltration of regulatory T cells into the tumor microenvironment may result in immunological resistance to targeted anti-VEGF treatment. When protected by the tumor microenvironment, non-tumor stromal cells and cell stroma provide an optimal habitat for surviving tumor cells and favorable conditions for tumor recurrence and metastasis [41,42].

Even though 95% of the genes in the human genome do not code for proteins, they do transcribe non-coding RNAs. Non-coding RNAs may be classified as housekeeping or regulatory RNAs based on their expression and functional properties. The former is essential for cell life activities and has a somewhat stable composition, enabling component expression. The latter has a high degree of selectivity, is often transiently expressed, and works as a transcriptional and translational regulator, affecting tumor treatment resistance. Among them, the association between long non-coding RNAs (lncRNAs), short non-coding RNAs (miRNAs), and circular non-coding RNAs (circRNAs) and tumor treatment resistance is gaining in attention. LncRNAs contribute to DNA repair and cell-cycle progression, drug metabolism and efflux, cell death, and EMT through encoding small-molecule polypeptides, contributing to tumor treatment resistance [43,44]. By augmenting the target gene’s mRNA with 6–7 bases and decreasing the target gene’s protein production level, miRNAs may confer resistance to malignancy therapy. Oncogenic miRNAs, in general, may reduce tumor suppressor gene expression, interfere with drug efferent pump molecules and cell cycle and apoptotic regulatory molecules, and ultimately result in tumor treatment resistance [45]. CircRNAs may function as an miRNA biomarker and therapeutic target for prognosis in malignancies via endogenous competition for miRNA and target mRNA binding sites or directly interact with proteins [46].

## 4. EMT-Induced Tumor Therapy Resistance

EMT was previously only associated with cancer cell invasion and metastasis. Recent research has shown that tumor cells undergoing EMT also have enhanced anti-apoptotic activity, which significantly contributes to tumor treatment resistance [47,48,49]. In addition, tumor cells that undergo EMT may acquire resistance to apoptosis, which directly influences the efficacy of radiation, chemotherapy, targeted therapy, and immunotherapy. Reversing EMT or eradicating tumor cells with EMT abnormalities has been proposed as a potential tumor therapy strategy [50].

### 4.1. EMT-Mediated Tumor Chemotherapy Resistance

Chemotherapy is a regularly utilized treatment approach for malignant tumors, although tumor cell resistance to chemotherapy drugs usually results in chemotherapy failure. Chemotherapy resistance mechanisms in tumor cells have previously been linked to cell cycle and phase specificity, drug uptake and efflux mechanisms, intracellular target substance concentrations, structural modifications, and enhanced DNA damage repair [51,52,53]. EMT has garnered significant attention as a potential mechanism of chemotherapeutic resistance in recent years due to its ability to turn tumor cells into tumor stem cells and confer resistance on tumor cells with resistance chemotherapy [54,55]. Without therapy, tumors are composed of clusters of chemically resistant cells that are dormant, dry, and well-coordinated in their EMT. These cells become more numerous during drug treatments and ultimately acquire resistance to chemotherapy. Similarly, EMT-mediated tumor phenotypic plasticity greatly influences resistance to chemotherapy in cancers [17].

Additionally, incomplete EMT or mesenchymal cells raised cancer stem cell markers and lowered the sensitivity to FAK inhibitors in MDA-MB-468 tumors treated with paclitaxel-nilotinib. Similar tumor stem cell markers were elevated in patients with metastatic prostate cancer treated with the PARP inhibitor tarazoparil [56,57]. Thus, the phenotypic plasticity conferred to tumor cells by EMT may result in tumor cells quickly responding to cytotoxicity or targeted molecular therapy, resulting in acquired drug resistance. By inhibiting FOXM1-mediated EMT and DNA repair, the expression of miR-671-5p dynamically decreases during the oncogenic transition of breast cancer. Therefore, miR-671-5p may be a promising biomarker for early breast cancer detection and a therapeutic target for breast cancer treatment [58].

**Figure 1:**
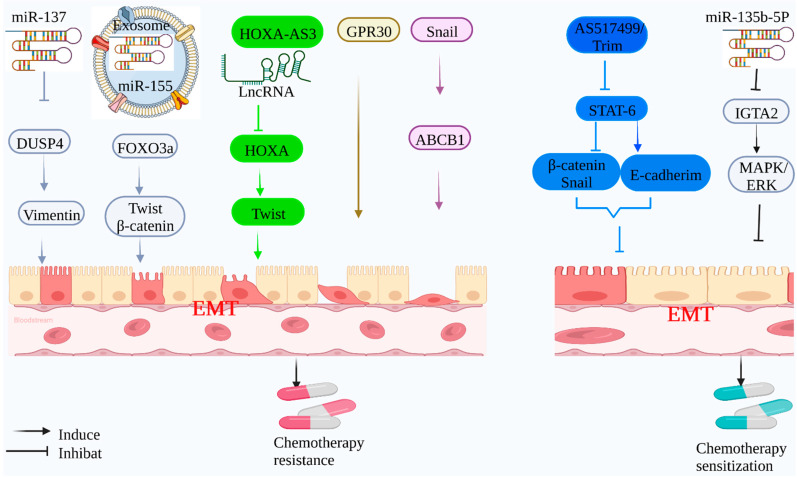
**Mechanism of EMT-mediated chemotherapy resistance:** Tumor-associated genes can induce EMT to mediate chemotherapy resistance of tumor cells. Mir-137 can inhibit the expression of target protein DUSP4 and induce EMT, which activates vimentin; exosomal Mir-155 can target the expression of FOXO3a and up-regulate the expression of β-catenin and Twist; lncRNA HOXA-AS3 can inhibit the expression of HOXA3 and up-regulate the expression of Twist, as well as participating in mediating adriamycin and cisplatin drug resistance; tumor-associated protein GPR30 can induce EMT and mediate cisplatin resistance. The tumor-related protein Snail regulates ABCB1 expression, induces EMT, and mediates 5-FU resistance. At the same time, 5-FU combined with AS1517499 and Trim inhibited the phosphorylation of STAT-6, up-regulated the expression of E-cadherin, and down-regulated the expression of Snail and β-catenin, delaying the progression of EMT, and enhanced the sensitivity of 5-FU to EMT-mediated chemotherapy resistance. Mir-135b-5p can target the expression of IGTA2, down-regulate the MAPK/ERK signaling pathway, delay the progression of EMT, and enhance the sensitivity of 5-FU.

Cisplatin is commonly used as a chemotherapeutic agent for oral squamous cell carcinoma. It was reported that EMT plays a significant role in the development of resistance to cisplatin therapy in oral squamous cell carcinoma. Mir-155, an exocrine compound, induced an EMT-mediated drug resistance phenotype in cisplatin-sensitive oral squamous cell carcinoma cells [59]. Through the induction of EMT, aberrant LncRNA expression may result in chemotherapeutic resistance. The lncRNA HOXA-AS3 induces EMT in NSCLC cells by targeting HOXA3 and increasing Twist expression [60]. Similarly, cisplatin treatment for gastric cancer may activate the estrogen receptor GPR30, leading to gastric cancer cells developing resistance to cisplatin through EMT [61,62]. In addition, EMT is inextricably connected to chemotherapy resistance in colorectal cancer. Snail, a molecule involved in EMT regulation, was significantly elevated in colorectal cancer tissues. The direct control of ABCB1 increased tumor cell resistance to 5-FU, indicating a probable relationship between EMT and treatment resistance [63] (Figure 1).

Given the crucial role of EMT in tumor chemotherapy resistance, it is desirable to target EMT and reverse it to MET to improve chemotherapy efficacy. 5-FU is the main treatment for colorectal cancer, and its combination with other adjuvant therapies (AS1517499 and Trim) has been demonstrated to enhance chemotherapy efficacy. When combined with 5-FU, this adjuvant treatment lowered STAT-6 phosphorylation, raised epithelial marker E-cadherin expression, decreased mesenchymal marker β-catenin and Snail1 expression, induced apoptosis, and enhanced 5-FU sensitivity [64]. The mir-135B-5p/ITGA2 signaling axis has been demonstrated to reduce the degree of EMT in gastric cancer chemotherapy trials by inhibiting the MAPK/ERK pathway, restoring gastric cancer cells’ chemotherapeutic sensitivity, and boosting mortality [65].

### 4.2. Tumor Radiation Resistance Mediated by EMT

Tumor radiotherapy is a strategy for locally treating tumor lesions with radiation, mainly killing or weakening tumor cells by inducing apoptosis and mitotic instability. Radiation resistance in tumors is a sophisticated process involving several genes and pathways that are tightly tied to cell cycle arrest, apoptosis resistance, tumor microenvironment changes, autophagy, and EMT, among other factors [66,67,68]. EMT-induced local recurrences and metastasis are critical causes of radiation failure. Numerous studies have shown a relationship between ionizing radiation-induced EMT in tumor cells, drug resistance in tumor cells, and the formation of tumor stem cells, although the process is not entirely known at the time [69,70].

EMT is intrinsically connected to tumor radiation resistance, and as EMT proceeds, tumor radiation sensitivity gradually decreases. TRIP4 overexpression in cervical cancer cells and tumor tissues may promote EMT and activate the PI3K/Akt and MAPK/ERK signaling pathways, enhancing radiation resistance in cervical cancer [71]. Radiation resistance is a frequent cause of the recurrence of glioblastoma. CXCL1 overexpression induces the mesenchymal transition and imparts radiation resistance to glioblastoma cells by stimulating the NF-B signaling pathway in glioblastoma cells [72].

Similarly, it was reported that EVI1, which is overexpressed in Nasopharyngeal carcinoma, could bind to Snail and HDAC1 to suppress E-cadherin synthesis and thus enhance the EMT of nasopharyngeal cancer cells. Meanwhile, EVI1 may directly interact with the promoter of β-catenin, boosting the stem cell properties of nasopharyngeal carcinoma (NPC) cells. EVI1 plays a role in nasopharyngeal cancer patients’ radiation resistance [73]. Individuals with recurrent nasopharyngeal cancer had more distant metastases and were more likely to develop radiation resistance after a second treatment than those with first diagnosed nasopharyngeal cancer. CCL2 expression was significantly increased in HONe1-IR cells and recurrent NPC tumors and contributed to radiation resistance through EMT. Additionally, inhibiting the mitochondrial pyruvate carrier (MPC) has been shown to promote EMT and confer radiation resistance in pancreatic and colorectal cancer cells [74].

**Figure 2:**
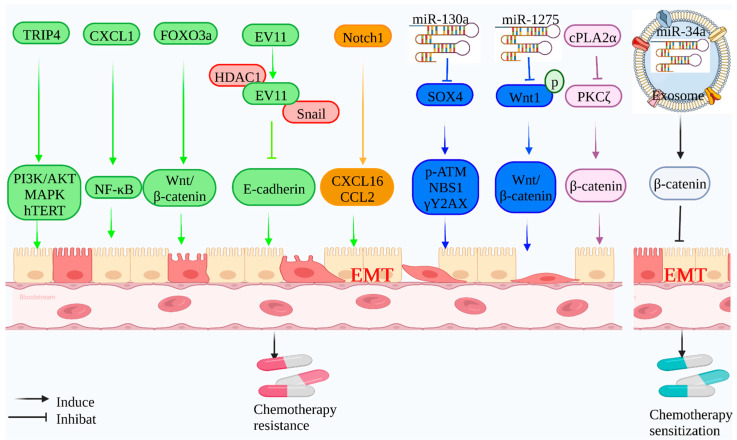
**Mechanism of EMT-mediated radiotherapy resistance:** Tumor-associated genes can mediate the radiation resistance of tumor cells by inducing EMT. Tumor-related proteins (TRIP4, CXCL1, FOXO3a, Notch1, and cPLA2α) can induce EMT through related signaling pathways (PI3K/Akt, NF-κB, Wnt/β-catenin, CCL2/CXCL16, and PKCζ/β-catenin) and enhance the radiotherapy tolerance of cervical cancer, glioblastoma, and nasopharyngeal carcinoma. EVI1 inhibits E-cadherin expression and induces EMT by forming a co-inhibitory complex with Snail and HDAC1 and enhances the radiotherapy tolerance of nasopharyngeal carcinoma. Tumor-associated non-coding RNAs (Mir-130a and Mir-1275) inhibit target proteins (SOX4 and WNT1), further influencing related proteins (NBS1, P-ATM, and γH2AX) and related signaling pathways (Wnt/β-catenin) to induce EMT and enhance the radiotherapy sensitivity of colorectal cancer and esophageal cancer. Meanwhile, in response to EMT-mediated radiotherapy resistance, exosome Mir-34A can target the expression of β-catenin, delay the progression of EMT, and improve the radiotherapy sensitivity of NPC.

Radiation resistance is inextricably linked to EMT and DNA damage repair in advanced colorectal cancer, and mir-130a may play a role in the radiation resistance mechanism in advanced colorectal cancer [75]. Similarly, mir-1275 improves esophageal cancer (EC) cells’ susceptibility to irradiation by suppressing EMT. Nonetheless, mir-1275 expression was down-regulated in the radiation-resistant esophageal cancer cell line KYSE-150R, and EMT was seen, leading to increased radiotherapy resistance in esophageal cancer cells [76] (Figure 2).

Inhibiting or reversing EMT may increase the radiosensitivity of cancers. Radiotherapy resistance in cervical cancer is intimately linked to cervical cancer stem cells. cPLA2 was discovered to regulate the reversible transition of cervical cancer stem cells between mesenchymal and epithelial states via the atypical protein kinase PKCζ, regulate cancer cell EMT state changes, and maintain various embryonic stem cells cell characteristics via the interaction between β-catenin and E-cadherin [77]. Consequently, EMT targeting cPLA2α to promote cervical cancer stem cells is a unique approach to radiosensitization therapy. Additionally, the exosome delivery of exogenous mir-34C to nasopharyngeal cancer may help to reduce EMT progression and increase radiation sensitivity [78].

### 4.3. EMT-Mediated Tumor-Targeted Therapy Resistance

With the rapid growth in molecular biology in cancer, molecular targeted therapy has been used in clinical practice and has gained growing importance in cancer medical treatment. While novel targets are rapidly being discovered, drug resistance remains a serious hurdle to the development of targeted therapies [79]. Cancer molecular targeted therapy is directed against tumor cells’ marker molecules in order to intervene in their carcinogenesis, such as inhibiting tumor cell proliferation, interfering with the cell cycle, inducing tumor cell differentiation and apoptosis, and inhibiting tumor cell metastasis and tumor vascular survival, to accomplish the purpose of tumor therapy. EMT may lead to drug resistance in tumor cells by modifying their behavior and function (for example, by promoting tumor spread, increasing drug efflux, or decreasing apoptotic signals), which is a crucial factor in drug resistance to targeted therapy [80,81].

At present, Gefitinib is the first-line therapeutic choice for patients with non-small cell lung cancer (NSCLC) who have an EGFR mutation. Gefitinib can promote tumor cell death and angiogenesis inhibition, but it may also delay the establishment of EMT in lung cancer cells by altering the link between HOTAIR and mir-34A-5p, hence exerting an additional effect antitumor activity [82]. This suggests that EMT may be closely related to the efficacy of EGFR-Tyrosine kinase inhibitors (EGFR-TKI). EMT is often associated with resistance to Gefitinib in lung cancer cells. By boosting interleukin-6 (IL-6) production in gefitinib-resistant non-small cell lung cancer cells, highly expressed LINC00460 acts as a competitive bait for mir-149-5p. Oct4 and Nanog coexpression affects drug resistance and EMT phenotypes by stimulating the Wnt/-βcatenin signaling pathway [83]. TGF-β1 overexpression has been demonstrated to promote ITG3 expression in patients with acquired Gefitinib or Oxitinib-resistant lung cancer. Antagonistic ITG3 inhibits lung cancer cell proliferation and EMT phenotypes, hence enhancing EGFR-TKI sensitivity [84]. Similar results were seen in gefitinib-resistant cell lines (HCC827GR and PC 9GR), where TGF-β1 -induced EMT contributed to gefitinib resistance through the mir-625-3p/AXL axis, and re-expression of the mir-625-3p partially restored gefitinib resistance. This further contributes to our knowledge of EGFR-TKI resistance [85].

Cetuximab, a monoclonal antibody directed against the EGFR, has been widely used to treat metastatic colorectal cancer; nevertheless, many patients who initially respond to cetuximab develop resistance [86]. Mir-141-3p participates in EMT by modulating the expression of E-cadherin, N-cadherin, Snail, and Vimentin. It also enhances the sensitivity of colorectal cancer cells to cetuximab by suppressing EGFR expression [87]. Meanwhile, RAS mutations limit the effectiveness of anti-EGFR monoclonal antibodies in patients receiving chemotherapy for metastatic colorectal cancer [88].

Curcumin is a plant polyphenol derived from the rhizome of turmeric, and its antitumor effect has been shown in liver cancer, lung cancer, and other malignant tumors. EMT is a critical biological link in the pathological process of esophageal cancer; it regulates the migration and invasion of cancer cells and has an indirect effect on the proliferation of cancer cells, chemotherapy resistance, and other biological behaviors [89]. During EMT, the epithelial phenotype is diminished along with the expression of the marker gene E-cadherin and cell polarity, enhancement of interstitial phenotype, increased expression of marker genes N-cadherin and Vimentin, and increased cell motility. Similarly, curcumin may enhance the expression of epithelial marker gene E-cadherin and reduce the expression of interstitial marker gene N-cadherin Vimentin; the greater the dosage of curcumin, the more pronounced the impact on gene expression regulation. These findings indicate that curcumin may greatly prevent EMT in esophageal cancer cells. EMT occurs in various malignant tumor lesions and is controlled by Notch and Wnt signaling pathways, according to publication number [90]. Notch1, Jagged1, Hes, Wnt1, and β-catenin were also reduced by curcumin in esophageal cancer cells. The Notch and Wnt pathway genes were evaluated after treatment with various dosages of curcumin, which increased the expression of GSK-3. These findings indicate that curcumin can greatly limit the activation of Notch and Wnt pathways in esophageal cancer cells and that curcumin may impede EMT by inhibiting Notch and Wnt pathways.

According to studies, resveratrol may reduce tumor invasion and metastasis by blocking EMT-related signaling pathways [91]. Resveratrol was reported to suppress the TGF-β-induced EMT process in lung cancer A549 cells by raising the expression of E-cadherin and lowering the expression of fibronectin, vimentin, and EMT-induced transcription factors Snail and Slug, consequently preventing cancer cell metastasis [92]. Likewise, Resveratrol suppresses TGF-β-induced EMT in colorectal cancer via reducing Smad protein, consequently limiting the invasion and metastasis of colorectal cancer cells. EMT is also proliferative vitreoretinopathy in retinal pigment epithelial cells. Important aspects of the pathogenesis Resveratrol may deacetylate Smad protein, hence reducing the EMT process generated by TGF-β and preventing proliferative vitreoretinopathy [93,94]. Table 1 below summarizes some molecular mechanisms by which EMT mediates cancer therapeutic resistance.

### 4.4. EMT-Mediated Tumor Immunotherapy Resistance

**Figure 3:**
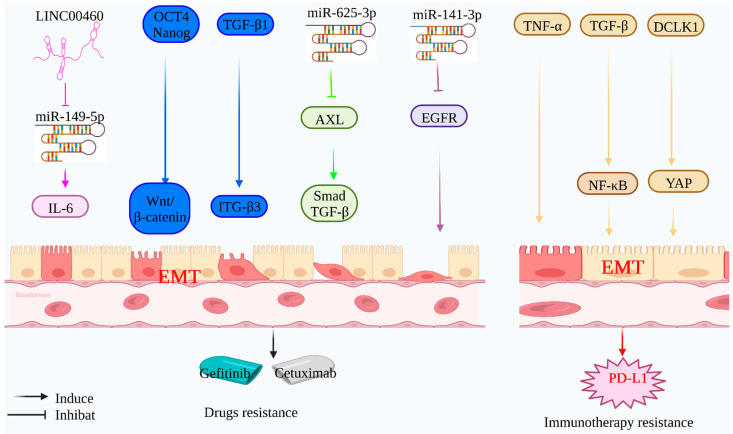
**Mechanism of EMT-mediated targeting and immunotherapy resistance:** Tumor-associated genes can induce EMT-mediated tumor cell targeting and immunotherapy resistance. LINC00460 can competitively inhibit the expression of Mir-149-5p and induce EMT by regulating the expression of IL-6, thus mediating gefitinib resistance. Mir-625-3p inhibited the expression of target gene AXL and activated the TGF-β/Smad signaling pathway to induce EMT. Mir-141-3p can inhibit the expression of target gene EGFR and induce EMT, thus mediating the resistance of gefitinib and cetuximab. Tumor-associated proteins (Oct4, Nanog, and TGF-β1) can induce EMT through related signaling pathways (Wnt/β-catenin and ITGβ3) and mediate gefitinib resistance. Tumor-associated proteins (TNF-α, TGF-β1, and DCLK1) can induce EMT through related signaling pathways (NF-κB and YAP), thereby mediating immunotherapy resistance.

Immune checkpoint inhibition is a significant advancement in tumor immunotherapy and is effective against a broad spectrum of advanced malignant tumors. However, low response rates and treatment resistance are essential hurdles to the advancement of immunotherapy. EMT has been associated with activating various immune checkpoint molecules, including programmed cell death ligand 1 (PD-L1) [97]. Thus, further exploration of the regulatory relationship between EMT and immune checkpoint molecules will improve immunotherapy’s sensitivity (Figure 3).

EMT endows tumor cells with the potential to avoid the immune system, which is controlled by immune checkpoint molecules in the majority of malignancies. This demonstrates a possible link between EMT and immunological checkpoint molecules. In hepatoma cell lines Hep3B and PLC/PRF/5, TNF-induced EMT increased the expression of PD-L1, PD-L2, CD73, and B7-H3, while reversing EMT reduced the expression of PD-L1, PD-L2, CD73, and B7-H3. Additionally, TNF was linked with a rise in PD-L1 expression in 422 HCC patients [96]. This study establishes a substantial link between the expression of immune checkpoint molecules and TNF-α-induced EMT. Similar incidences of gastric and pancreatic cancer have been reported. EMT may enhance the migration and invasion potential of gastric cancer cells, depending on the amount to which PD-L1 expression is enhanced due to NF-кB activation [95]. DCLK1 is integrally engaged in tumor cell EMT and may also regulate the expression of PD-L1 through the Hippo pathway-associated protein (YAP) [98].

Regulating EMT and the tumor immune response may be a potential strategy for increasing the efficacy of immunotherapy. CMTM6 is a PD-L1 regulatory molecule, and it has been shown that silencing CMTM6 decreases PD-L1 expression and increases CD8+ and CD4+ T cell infiltration. Meanwhile, via the Wnt/β-catenin signaling pathway, CMTM6 may increase tumor cell dehydration and EMT [99]. CMTM6 may be a potential target for immune sensitization therapy due to its capacity to simultaneously regulate tumor cell dryness, EMT, and immune response. REDOX states are present in melanoma and non-small cell lung cancer cell lines and are regulated by numerous aldehyde dehydrogenases (ALDHs). ALDH3A1 has been demonstrated to trigger EMT in melanoma and non-small cell lung cancer cells, while the overexpression of ALDH3A1 enhances PD-L1 expression and lowers monocyte proliferation in peripheral blood [90]. These results imply that ALDH3A1 is inextricably linked to EMT and PD-L1 and regulates tumor immune output.

## 5. Therapeutic Strategies for EMT-Mediated Tumor Therapeutic Resistance

Given the crucial role of EMT in tumor resistance, reversing or inhibiting EMT represents a unique idea for therapeutic tumor sensitization. MET is thought to be the diametric opposite of EMT. It leads to the suppression of mesenchymal characteristics of tumor cells and the re-expression of epithelial markers, which improves tumor cells’ resistance to therapy [100,101,102]. The conversion of mesenchymal tumor cells to an epithelioid phenotype may provide a unique method for cancer treatment resistance.

MET may be formed due to the reduced expression of EMT transcription factors (EMT-TRAN and transcription of EMT-TFS), and targeting EMT-TFS may be a strategy for treating malignancies that have developed resistance to treatment [103]. Ionizing radiation has been found to promote EMT and increase the radiation resistance of hypopharyngeal cancer cells through the AKT/GSK-3/Snail signaling pathway. However, silencing Snail reverses EMT and significantly lowers the radiation resistance of hypopharyngeal cancer cells [104]. It has been shown that silencing Slug increases cancer cells’ radiation sensitivity in oral squamous cell carcinoma [105]. These results suggest that Snail and Slug may be potential EMT-mediated radiation resistance therapy targets. Twist may form a dimer or heterodimer with the E-cadherin promoter region, inhibiting its expression and promoting the initiation of EMT [106]. When sitwist-MSN-HA, a novel nanoparticle delivery platform, interfered with Twist expression, naked mice were more vulnerable to cisplatin in epithelial ovarian cancer, showing that the technology may have been exploited in malignancies with the overexpression of Twist [107]. Not only is ZEB1 required for EMT activation, but it also plays a crucial role in the development of chemotherapeutic resistance. Interfering with ZEB1 expression has been demonstrated to increase the sensitivity of colorectal cancer cells to chemotherapy, suggesting that reducing ZEB1 expression may help restore sensitivity in ZEB1-mediated chemotherapy resistance [108].

Numerous signaling pathways are implicated in tumor cell EMT, and inhibitors of these signaling pathways may effectively delay EMT to a certain extent, hence diminishing tumor resistance to treatment. TGF-β/Smad signaling is a well-characterized signaling mechanism that regulates EMT. The TGF-receptor inhibitors LV2109761 and LV364947 have been demonstrated to block EMT generated by ionizing radiation in gastric cancer and glioma cells, hence boosting tumor cells’ irradiation sensitivity [109,110,111]. Additionally, tumor EMT and treatment resistance are related to the Wnt/β-catenin signaling pathway [112]. Indeed, the overexpression of the Wnt/β-catenin signaling pathway has been demonstrated to promote tumor cell EMT and resistance to treatment. WNT974 inhibits the Wnt/β-catenin signaling system, which may significantly increase lymphoma chemotherapeutic sensitivity to driamycin. The inhibitor XAV939 has dramatically increased cervical cancer cells’ radiation sensitivity [113,114].

Several recent studies demonstrated a correlation between EMT and the regulation of certain ribosomal proteins in various forms of cancer. Some ribosomal proteins have shown the potential to regulate cell migration, altering the EMT process and eventually resulting in chemoresistance [115,116]. The down-regulation of ribosomal protein uL3 is associated with increased cell migration and an EMT resulting in chemoresistance, while the silencing of ribosomal protein RPL34 is sufficient to reduce the EMT phenotype, preventing esophageal cancer cell migration and invasion [117,118]. The translation of ribosomal protein-coding mRNAs (RP-mRNAs) causes the creation of ribosomes; nevertheless, the mechanisms that regulate RP-mRNA translation in conjunction with other cellular activities are not well understood. The RP-mRNAs are localized in actin-rich cell protrusions during cell migration into their surroundings. RNA-binding protein La-related protein 6 is responsible for this localization (LARP6). Cell migration depends on LARP6-mediated mRNA localization, and this pathway is related to cancer development during EMT [116,119].

## 6. Outlooks

The mechanism by which tumor cells develop treatment resistance is complex, and therapeutic efficiency and resistance processes differ significantly across individuals with diverse tumors. The heterogeneity and biological mechanisms such as EMT, autophagy, cell cycle arrest, DNA damage repair, drug metabolism, and efflux all contribute to tumor treatment resistance. Additionally, the tumor microenvironment and other small-molecule compounds in the circulatory system may contribute to the resistance management of tumor therapy. However, intervening in only one of the steps outlined above is insufficient to completely reverse tumor cell treatment resistance: future research should focus on the underlying mechanism of tumor cells from the treatment-resistant phenotype. EMT is one of the most common mechanisms underlying resistance to cancer treatment. As research has advanced, the role of EMT and the molecular mechanism by which EMT occurs have become well understood. Numerous clinical studies have shown a clear link between EMT, chemotherapy tolerance, and radiation resistance in cancer patients. However, there is currently a shortage of therapeutic drugs or treatment techniques that may boost the EMT process and hence the efficiency of tumor therapy. Although cell and animal studies have shown that targeting specific locations may reverse EMT and boost tumor therapy sensitivity, there are still considerable risks and obstacles to long-term human success. As a result, a more comprehensive and detailed study of the relationship between EMT and tumor treatment resistance is necessary. It is still necessary to coordinate basic experiments, clinical research, and development with translational medicine at the core to develop a new plan for personalized tumor treatment in the future.

## Figures and Tables

**Table 1:** EMT-mediated therapeutic resistance.

Tumor Types	Mechanisms	References
**Breast**	Inhibition of EMT and chemoresistance in breast cancer cells by miR-137.	[19]
**Gastric**	Cisplatin treatment of AGS and BGC-823 resulted in EMT. EMT enhances the ability of gastric cancer cells to migrate and invade	[61,95]
**Cervical**	TRIP4 depletion dramatically reduced cervical tumor cell proliferation and EMT	[71]
**Colorectal**	Snail, an EMT regulator, may induce chemoresistance by boosting the activity of the ABC transporter ABCB1. After exposure to ionizing radiation, rectal cancer cells displayed an EMT transition phenotype, which was reversed when miR-130a suppressed cell invasion.	[63,75]
**Esophageal**	miR-1275 specifically targeted WNT1, thereby inhibiting the Wnt/β-catenin signaling pathway in Esophageal cancer cells via EMT.	[76]
**Glioblastoma**	Inhibiting CXCL1 expression decreased the growth and radioresistance of Glioblastoma cells.	[72]
**Nasopharyngeal**	Increased EVI1 expression in Nasopharyngeal cells was related to a poor prognosis and was shown to cause chemo/radioresistance in these cells.	[73]
**Liver**	Overexpression of TNF-α and PD-L1 in Hepatocellular Carcinoma was related to a poor overall survival outcome.	[96]
**Lung**	HOXA-AS3 conferred resistance to cisplatin by downregulating homeobox A3 expression (HOXA3). Cisplatin resistance was also increased, as was the EMT induced by HOXA3 knockdown. Oct4/Nanog stimulation of the Wnt/β-catenin signaling pathway controls drug resistance and EMT changes. Inhibition of β-catenin abolished the multi-drug resistance and EMT processes mediated by Oct4/Nanog in lung cancer cells.	[60,83]

## Data Availability

Not applicable.
